# Supplementary material for: Cultural and contextual adaptation of mental health measures in Kenya: An adolescent-centered transcultural adaptation of measures study
Source: PLoS One. 2022 Dec 15;17(12):e0277619. doi: 10.1371/journal.pone.0277619 (PMC9754261; doi:10.1371/journal.pone.0277619)
Supplement: S1 Checklist — (DOC) [file pone.0277619.s001.doc]

**Consolidated criteria for reporting qualitative studies (COREQ): 32-item checklist**

Developed from:

Tong A, Sainsbury P, Craig J. Consolidated criteria for reporting qualitative research (COREQ): a 32-item checklist for interviews and focus groups. *International Journal for Quality in Health Care*. 2007. Volume 19, Number 6: pp. 349 – 357

| **No.  Item** | **Guide questions/description** | **Response** |
| --- | --- | --- |
| **Domain 1: Research team and reﬂexivity** |  |  |
| *Personal Characteristics* |  |  |
| 1. Inter viewer/facilitator | Which author/s conducted the interview or focus group? | SM, VN, JK, OY, MK |
| 2. Credentials | What were the researcher’s credentials? E.g. PhD, MD | MSc, BSc, MSc, PhD, PhD, |
| 3. Occupation | What was their occupation at the time of the study? | Clinical psychologist, Research assistant, Clinical psychologist, Clinical psychologist, Senior lecturer |
| 4. Gender | Was the researcher male or female? | Both |
| 5. Experience and training | What experience or training did the researcher have? | MK, has PhD degree and have supervised several PhD and Masters level students in conducting both quantitative and qualitative research. OY has PhD with experience in adolescent mental health research. VN, JK, SM are research associates with experience in conducting qualitative research |
| *Relationship with participants* |  |  |
| 6. Relationship established | Was a relationship established prior to study commencement? | None of the participants had an established relationship with any of the authors prior to study commencement. |
| 7. Participant knowledge of the interviewer | What did the participants know about the researcher? e.g. personal goals, reasons for doing the research | Participants were informed that the researchers were interested in testing the their knowledge of mental health terms and common words used in their areas, to help in adapting tools to their context. |
| 8. Interviewer characteristics | What characteristics were reported about the inter viewer/facilitator? e.g. Bias, assumptions, reasons and interests in the research topic | A brief introduction about the study, organization and interviewer’s names and positions provided to the participants before starting the interview. Interviewer-related biases were not identified. |
| **Domain 2: study design** |  |  |
| *Theoretical framework* |  |  |
| 9. Methodological orientation and Theory | What methodological orientation was stated to underpin the study? e.g. grounded theory, discourse analysis, ethnography, phenomenology, content analysis | Thematic content analysis |
| *Participant selection* |  |  |
| 10. Sampling | How were participants selected? e.g. purposive, convenience, consecutive, snowball | Purposive non-probability sampling |
| 11. Method of approach | How were participants approached? e.g. face-to-face, telephone, mail, email | Face-to-face interviews were conducted. |
| 12. Sample size | How many participants were in the study? | 62 |
| 13. Non-participation | How many people refused to participate or dropped out? Reasons? | NA |
| *Setting* |  |  |
| 14. Setting of data collection | Where was the data collected? e.g. home, clinic, workplace | All interviews were conducted at 2 identified health facilities located in the community/study area. |
| 15. Presence of non-participants | Was anyone else present besides the participants and researchers? | No |
| 16. Description of sample | What are the important characteristics of the sample? e.g. demographic data, date | 42 adolescent participants ages 10-19 years old.  20 caregivers of adolescents ages 10- 14 years.  FGDs and cognitive interviews were conducted in November and December 2020 (During COVID-19 pandemic) |
| *Data collection* |  |  |
| 17. Interview guide | Were questions, prompts, guides provided by the authors? Was it pilot tested? | Interview guide was prepared was rigorously discussed among the authors. Probes were used to facilitate discussions. |
| 18. Repeat interviews | Were repeat inter views carried out? If yes, how many? | No |
| 19. Audio/visual recording | Did the research use audio or visual recording to collect the data? | Interviews were audio recorded. |
| 20. Field notes | Were ﬁeld notes made during and/or after the interview or focus group? | Field notes after the interview were also transcribed and translated and included in the dataset for the analysis. |
| 21. Duration | What was the duration of the inter views or focus group? | Focus group discussions lasted between 1- 2 hours, while cognitive interviews lasted for about 30-45 minutes. |
| 22. Data saturation | Was data saturation discussed? | Yes |
| 23. Transcripts returned | Were transcripts returned to participants for comment and/or correction? | No |
| **Domain 3: analysis and ﬁndings** |  |  |
| *Data analysis* |  |  |
| 24. Number of data coders | How many data coders coded the data? | Authors independently coded the data  (MK, DN, SM, OY, VN, JK) |
| 25. Description of the coding tree | Did authors provide a description of the coding tree? | Yes, the authors generated a coding system and codebook in MS Excel with domains, codes, and themes generated.  Yes, coders generated a coding framework, themes identified and a coding matrix developed |
| 26. Derivation of themes | Were themes identiﬁed in advance or derived from the data? | The analysis was integrated where by themes were identified inductively from the data (grounded) and also deductively (in advance guided by the interview question guide and the |
| 27. Software | What software, if applicable, was used to manage the data? | NVivo version 10 |
| 28. Participant checking | Did participants provide feedback on the ﬁndings? | No |
| *Reporting* |  |  |
| 29. Quotations presented | Were participant quotations presented to illustrate the themes/ﬁndings? Was each quotation identiﬁed? e.g. participant number | Yes, participants’ quotations were present to support each theme.  Yes, each quotation was identified by the unique participant number. |
| 30. Data and ﬁndings consistent | Was there consistency between the data presented and the ﬁndings? | Yes, there was consistency between the data presented and the findings. |
| 31. Clarity of major themes | Were major themes clearly presented in the ﬁndings? | Yes. Yes, major themes were clearly presented in the Results section as sub heading with supporting quotations |
| 32. Clarity of minor themes | Is there a description of diverse cases or discussion of minor themes? | Yes. Yes, minor themes were clearly presented in the Results section using specific sections regarding each theme. |
