# Supplementary material for: Cultural and contextual adaptation of mental health measures in Kenya: An adolescent-centered transcultural adaptation of measures study
Source: PLoS One. 2022 Dec 15;17(12):e0277619. doi: 10.1371/journal.pone.0277619 (PMC9754261; doi:10.1371/journal.pone.0277619)
Supplement: S1 Table — (DOCX) [file pone.0277619.s002.docx]

**Supporting Information**

**STable 1 Item adjustment Annex**

|  | **Original RCADS items** | **Simplification to Kenyan English** | **Kiswahili translation** | **English version adjusted after FGDs** | **Kiswahili version adjusted after FGDs** | **Final English version after CIs and expert review** | **Final Kiswahili version after CIs and expert review** |
| --- | --- | --- | --- | --- | --- | --- | --- |
| **Revised Children’s Anxiety and Depression Scale (RCADS)** | | | | | | | |
| AD3 | I feel sad or empty… | I feel sad … | Nahisi huzuni … | I feel sad … | Nahisi huzuni … | I feel sad … | Nasikia huzuni … |
| AD4 | I worry when I think I have done poorly at something… | I worry when I think I have done something poorly… | Ninakuwa na wasiwasi ninapofikiri nimefanya kitu vibaya… | I worry when I think I have done something poorly… | Ninakuwa na wasiwasi ninapofikiri nimefanya kitu vibaya… | I worry when I think I have done something poorly… | Nina wasiwasi ninapofikiri nimefanya kitu vibaya… |
| AD5 | I would feel afraid of being on my own at home… | I would feel afraid of being alone at home… | Ningehisi uoga kubaki nyumbani peke yangu… | I would feel afraid of being alone at home… | Ningesikia uoga kubaki nyumbani peke yangu… | I would feel afraid of being alone at home… | Ningesikia uwoga kubaki nyumbani peke yangu… |
| AD6 | Nothing is much fun anymore… | Nothing is much fun anymore… | Hakuna kitu kinachofurahisha tena… | Nothing is much fun anymore… | Hakuna kitu kinachofurahisha tena… | Nothing is much fun anymore… | Hakuna kitu kinachofurahisha tena… |
| AD7 | I feel scared when I have to take a test… | I feel scared when I have to take an exam… | Ninahisi uoga wakati ni lazima nifanye mtihani… | I feel scared when I have to take an exam… | Ninasikia uoga wakati ni lazima nifanye mtihani…/mimi naogopa wakati wa mtihani | I feel scared when I have to take an exam… | Mimi naogopa wakati wa mtihani… |
| AD8 | I feel worried when I think someone is angry with me… | I feel worried when I think someone is angry with me… | Nahisi wasiwasi wakati ninapodhania mtu amenikasirikia… | I feel worried when I think someone is angry with me… | Nahisi wasiwasi wakati ninapodhania mtu amenikasirikia… | I feel worried when I think someone is angry with me… | Nasikia wasiwasi wakati ninapofikiria mtu amenikasirikia… |
| AD9 | I worry about being away from my parents… | I worry about being away from my parents… | Napata wasiwasi wa kuwa mbali na wazazi wangu… | I worry about being away from my parents… | Napata wasiwasi wa kuwa mbali na wazazi wangu… | I worry about being away from my parents… | Napata wasiwasi wa kuwa mbali na wazazi wangu… |
| AD10 | I have trouble sleeping… | I have trouble sleeping… | Ninapata shida ya kulala… | I have trouble in sleeping… | Ninapata shida ya kulala… | I have trouble in sleeping… | Ninapata shida ya kulala… |
| AD11 | I worry that I will do badly at my school work… | I worry that I will not do well in my school work… | Nina wasiwasi kuwa sitofaulu katika kazi zangu za shule… | I worry that I will not do well in my school work… | Nina wasiwasi kuwa sitafanya vizuri katika masomo yangu ya shule… | I worry that I will not do well in my school work… | Nina wasiwasi kuwa sitafanya vizuri katika masomo yangu ya shule… |
| AD12 | I worry that something awful will happen to someone in my family… | I worry that something bad will happen to someone in my family… | Nina wasiwasi kuwa kitu kibaya kitamfanyikia mtu wa familia yangu… | I worry that something bad will happen to someone in my family… | Nina wasiwasi kuwa kitu kibaya kitamfanyikia mtu wa familia yangu… | I worry that something bad will happen to someone in my family… | Nina wasiwasi kuwa kitu kibaya kitamfanyikia mtu wa familia yangu… |
| AD13 | I suddenly feel as if I can't breathe when there is no reason for this… | I suddenly feel as if I can't breathe when there is no reason for this… | Ghafla nahisi kana kwamba siwezi pumua, pasipo na sababu… | I suddenly feel as if I can't breathe when there is no reason for this… | Ghafla nahisi kama siwezi pumua, pasipo na sababu… | I feel as if I can't breathe without a reason… | Nasikia kama siwezi pumua bila sababu… |
| AD14 | I have problems with my appetite… | I have problems with my appetite… | Ninashida ya hamu ya kula… | I have problems with my appetite… | Ninashida ya hamu ya kula chakula… | I have problems with my appetite for food… | Nina shida ya hamu ya kula chakula… |
| AD15 | I feel scared if I have to sleep on my own… | I feel scared if I have to sleep alone… | Nahisi uoga ninapolazimika kulala peke yangu… | I feel scared if I have to sleep alone… | Nahisi uoga ninapolazimika kulala peke yangu… | I feel scared if I have to sleep alone… | Nasikia uwoga ninapolazimika kulala peke yangu… |
| AD16 | I have no energy for things… | I have no energy for anything… | Sina nguvu za kufanya chochote… | I have no energy for anything… | Sina nguvu za kufanya chochote… | I have no energy for anything… | Sina nguvu za kufanya chochote… |
| AD17 | I worry I might look foolish… | I worry I might look foolish… | Nina wasiwasi ninaweza onekana mjinga… | I worry I might look foolish… | Nina wasiwasi ninaweza onekana mjinga… | I worry I might look foolish… | Nina wasiwasi ninaweza onekana mjinga… |
| AD18 | I am tired a lot… | I am tired a lot… | Mimi huchoka sana… | I am tired a lot… | Mimi huchoka sana… | I am tired a lot… | Mimi huchoka sana… |
| AD19 | I worry that bad things will happen to me… | I worry that bad things will happen to me… | Nina wasiwasi wa kutokewa na mambo mabaya… | I worry that bad things will happen to me… | Nina wasiwasi wa kutokewa na mambo mabaya… | I worry that bad things will happen to me… | Nina wasiwasi wa kufanyikiwa na mambo mabaya… |
| AD20 | I cannot think clearly… | I cannot think clearly… | Siwezi fikiria vizuri/waziwazi… | I cannot think clearly… | Siwezi fikiria vizuri/waziwazi… | I cannot think very well… | Siwezi fikiria vizuri… |
| AD21 | I suddenly start to tremble or shake when there is no reason for this… | I suddenly start to tremble or shake when there is no reason for this… | Ghafla mimi huanza kutetemeka bila sababu… | I suddenly start to tremble or shake when there is no reason for this… | Ghafla mimi huanza kutetemeka bila sababu… | I start to tremble or shake without a reason… | Mimi huanza kutetemeka bila sababu… |
| AD22 | I worry that something bad will happen to me… | I worry that something bad will happen to me… | Nina wasiwasi kitu kibaya kitanifanyikia/ kunitokea… | I worry that something bad will happen to me… | Nina wasiwasi kitu kibaya kitanifanyikia/ kunitokea… | I worry that something bad will happen to me… | Nina wasiwasi kitu kibaya kitanifanyikia… |
| AD23 | When I have a problem, I feel shaky… | when I have a problem, I feel shaky… | Ninapokuwa na shida, nahisi kutetemeka… | when I have a problem, I feel shaky… | Ninapokuwa na shida, nahisi kutetemeka… | when I have a problem, I feel shaky or tremble… | Ninapokuwa na shida, nasikia kutetemeka… |
| AD24 | I feel worthless… | I feel worthless… | Najihisi sina maana… | I feel worthless… | Najihisi sina maana… | I feel I have no meaning… | Nasikia sina maana… |
| AD25 | I worry about making mistakes… | I worry about making mistakes… | Ninakuwa na wasiwasi wa kufanya makosa… | I worry about making mistakes… | Ninakuwa na wasiwasi wa kufanya makosa… | I worry about making mistakes… | Nina wasiwasi wa kufanya makosa… |
| AD26 | I worry what other people think of me… | I worry what other people think of me… | Ninakuwa na wasiwasi kuhusu vipi watu wanavyonifikiria… | I worry what other people think of me… | Ninakuwa na wasiwasi kuhusu vipi watu wanavyonifikiria… | I worry what other people think of me… | Nina wasiwasi kuhusu vile watu wanavyonifikiria… |
| AD27 | I am afraid of being in crowded places (like shopping centers, the movies, buses, busy playgrounds)… | I am afraid of being in crowded places (like shopping centers, busy playgrounds, bus stations, busy streets, market places)… | Naogopa kuwa mahali penye watu wengi (kwenye maduka makuu, sinema, kituo cha basi, uwanja wa michezo wenye shughuli nyingi)… | I am afraid of being in crowded places (like shopping centers, busy playgrounds, bus stations, busy streets, market places)… | Naogopa kuwa mahali penye watu wengi (kwenye maduka makuu, uwanja wa michezo wenye shughuli nyingi, kituo cha basi, barabara zenye shughuli nyingi, sokoni)… | I am afraid of being in crowded places (like shopping centers, busy playgrounds, bus stations, busy streets, market places)… | Naogopa kuwa mahali penye watu wengi (kama kwenye maduka makuu, uwanja wa michezo wenye shughuli nyingi, kituo cha basi, barabara zenye shughuli nyingi, sokoni)… |
| AD28 | All of a sudden I feel really scared for no reason at all… | All of a sudden I feel really scared for no reason at all… | Kwa ghafla huhisi kuogopa sana bila sababu hata kidogo… | I feel really scared for no reason … | Mimi huhisi kuogopa sana bila sababu … | I feel really scared without a reason … | Mimi husikia kuogopa sana bila sababu … |
| AD29 | I worry about what is going to happen… | I worry about what is going to happen… | Nina wasiwasi kuhusu kitakachofanyika… | I worry about what is going to happen… | Nina wasiwasi kuhusu kitakachofanyika… | I worry about what is going to happen… | Nina wasiwasi kuhusu kitakachofanyika… |
| AD30 | I suddenly become dizzy or faint when there is no reason for this… | I suddenly become dizzy or faint when there is no reason for this… | Ghafla ninakuwa na kizunguzungu au kuzimia bila sababu yoyote… | I become dizzy or faint when there is no reason … | Ninakuwa na kizunguzungu au kuzimia bila sababu yoyote… | I become dizzy or faint without a reason … | Ninakuwa na kizunguzungu au kuzimia bila sababu… |
| AD31 | I think about death… | I think about death… | Ninafikiria kuhusu kifo… | I think about death… | Ninafikiria kuhusu kifo… | I think about death… | Ninafikiria kuhusu kifo… |
| AD32 | My heart suddenly starts to beat too quickly for no reason… | My heart suddenly starts to beat too quickly for no reason… | Ghafla roho yangu huanza kupiga kwa haraka bila sababu… | My heart suddenly starts to beat too quickly for no reason… | Ghafla roho yangu huanza kupiga kwa haraka bila sababu… | My heart starts to beat too quickly without a reason… | Roho yangu huanza kupiga kwa haraka bila sababu… |
| AD33 | I feel like I don’t want to move… | I feel like I don’t want to move… | Nahisi kama sitaki kusonga au kutingishika… | I feel like I don’t want to move… | Nahisi kama sitaki kusonga … | I feel like I don’t want to move… | Nasikia kama sitaki kusonga … |
| AD34 | I worry that I will suddenly get a scared feeling when there is nothing to be afraid of… | I worry that I will suddenly get a scared feeling when there is nothing to be afraid of… | Ninawasiwasi wa kupata hisia ya uoga ghafla bila sababu ya kuogofya… | I worry that I will suddenly get a scared feeling when there is nothing to be afraid of… | Ninawasiwasi wa kupata hisia ya uoga ghafla bila sababu ya kuogopa… | I worry that I will get a scared feeling when there is nothing to be afraid of… | Nina wasiwasi wa kupata fikira ya uwoga bila sababu ya kuogopa… |
| AD35 | I feel afraid that I will make a fool of myself in front of people… | I feel afraid that I will embarrass myself in front of people… | Nahisi uoga kuwa nitajifanya nionekane mjinga mbele za watu… | I feel afraid that I will shame myself in front of people… | Nahisi uoga kuwa nitajiaibisha mbele za watu… | I feel afraid that I will shame myself in front of people… | Nasikia uwoga kuwa nitajiaibisha mbele za watu… |
| AD36 | I would feel scared if I had to stay away from home overnight… | I would feel scared if I had to stay away from home overnight… | Ningehisi uwoga ikiwa itabidi nikae mbali na nyumbani usiku kucha… | I would feel scared if I had to stay away from home overnight… | Ningehisi uwoga ikiwa itabidi nikae mbali na nyumbani usiku wote… | I would feel scared if I had to stay away from home the whole night… | Ningesikia uwoga ikiwa itabidi nikae mbali na nyumbani usiku wote… |
| AD37 | I feel restless… | I feel restless… | Nahisi sina utulivu… | I feel restless… | Nahisi sina utulivu/amani… | I feel restless or not at peace… | Nasikia sina utulivu/amani… |
| FX3 | We have been talking about different feelings and experiences that may be a problem for people your age.  Point to the response card and say: please answer my questions using the same responses: always, often, sometimes, never. | we have been talking about different feelings and experiences that may be a problem for people your age.  Point to the response card and say: please answer my questions using the same responses: always, often, sometimes, never. | Tumekuwa tukiongea kuhusu hisia tofauti na mazoea/mambo watu wa umri wako wanayopitia ambayo yanaweza kuwatatiza.  Elekeza kidole kwenye kadi ya majibu na kisha useme: tafadhali jibu maswali yangu kutumia majibu kama yale ya kwanza: kila wakati, mara nyingi, wakati mwingine, sikupata kabisa. | we have been talking about different feelings and experiences that may be a problem for people your age.  Point to the response card and say: please answer my questions using the same responses: always, often, sometimes, never. | Tumekuwa tukiongea kuhusu hisia tofauti na mazoea/mambo watu wa umri wako wanayopitia ambayo yanaweza kuwatatiza.  Elekeza kidole kwenye kadi ya majibu na kisha useme: tafadhali jibu maswali yangu kutumia majibu kama yale ya kwanza: kila wakati, mara nyingi, wakati mwingine, sikupata kabisa. | we have been talking about different feelings and experiences that may be a problem for people your age.  Point to the response card and say: please answer my questions using the same responses: always, often, sometimes, never. | Tumekuwa tukiongea kuhusu hisia au fikira tofauti na mazoea/mambo watu wa umri wako wanayopitia ambayo yanaweza kuwatatiza au kuwasumbua.  Elekeza kidole kwenye kadi ya majibu na kisha useme: tafadhali jibu maswali yangu kutumia majibu kama yale ya kwanza: kila wakati, mara nyingi, wakati mwingine, sikupata kabisa. |
| FX4 | In the past month, how often did these problems interfere with your daily activities or relationships at home? | In the past month, how often did these problems interfere/disrupt your daily activities or relationships at home? | Katika mwezi uliopita, ni mara ngapi shida hizi zimekuwa zikitatiza kazi zako za kila siku na uhusiano nyumbani? | In the past month, how often did these problems interfere/disrupt your daily activities or relationships at home? | Katika mwezi uliopita, ni mara ngapi shida hizi zimekuwa zikitatiza kazi zako za kila siku na uhusiano nyumbani? | In the past month, how often did these problems interfere/disrupt your daily activities or relationships at home? | Katika mwezi uliopita, ni mara ngapi shida hizi zimekuwa zikitatiza au zikisumbua kazi zako za kila siku na uhusiano nyumbani? |
| FX5 | In the past month, how often did these problems interfere with your activities at school/work? | In the past month, how often did these problems interfere/disrupt your activities at school/work? | Katika mwezi uliopita, ni mara ngapi shida hizi zimekua zikitatiza shughuli zako za shuleni/kazini? | In the past month, how often did these problems interfere/disrupt your activities at school/work? | Katika mwezi uliopita, ni mara ngapi shida hizi zimekua zikitatiza shughuli zako za shuleni/kazini? | In the past month, how often did these problems interfere/disrupt your activities at school/work? | Katika mwezi uliopita, ni mara ngapi shida hizi zimekua zikitatiza au zikisumbua shughuli zako za shuleni/kazini? |
| FX6 | In the past month, how often did these problems cause any issues for you with your friends? | In the past month, how often did these problems cause any issues for you with your friends? | Katika mwezi uliopita, ni mara ngapi shida hizi zimesababisha maswala fulani/vurugu kati yako na marafiki zako? | In the past month, how often did these problems cause any issues for you with your friends? | Katika mwezi uliopita, ni mara ngapi shida hizi zimesababisha maswala fulani/vurugu kati yako na marafiki zako? | In the past month, how often did these problems cause any disagreements with your friends? | Katika mwezi uliopita, ni mara ngapi shida hizi zimesababisha kukosana au kutoelewana kati yako na marafiki zako? " |
| PS1 | From the start of this interview, we have been talking about different feelings, experiences and problems that people your age may go through.  When you have a problem or worry about these sorts of feelings and experiences, how often do you talk to someone else about it? | From the start of this interview, we have been talking about different feelings, experiences and problems that people your age may go through.  When you have a problem or worry about these types of feelings and experiences, how often do you talk to someone else about it? | Kuanzia mwanzo wa mahojiano haya, tumekuwa tukizungumuzia kuhusu hisia, uzoefu na shida tofauti ambazo watu wa rika lako wanaweza kupitia.  Wakati una shida kama hizi au wasiwasi kuhusu hisia tofauti na mambo ya aina yoyote unayopitia, ni mara ngapi unaongea na mtu mwingine kuyahusu? | From the start of this interview, we have been talking about different feelings, experiences and problems that people your age may go through.  When you have a problem or worry about these types of feelings and experiences, how often do you talk to someone else about it? | Kuanzia mwanzo wa mahojiano haya, tumekuwa tukizungumuzia kuhusu hisia, mazoea na shida tofauti ambazo watu wa rika lako wanaweza kupitia.  Wakati una shida kama hizi au wasiwasi kuhusu hisia tofauti na mambo ya aina yoyote unayopitia, ni mara ngapi unaongea na mtu mwingine kuyahusu? | From the start of this interview, we have been talking about different feelings, experiences and problems that people your age may go through.  When you have a problem or worry about these types of feelings and experiences, how often do you talk to someone else about it? | Kuanzia mwanzo wa mahojiano haya, tumekuwa tukiongelea kuhusu hisia au fikira, mazoea na shida tofauti ambazo watu wa rika lako wanaweza kupitia.  Wakati una shida kama hizi au wasiwasi kuhusu hisia au fikira tofauti na mambo ya aina yoyote unayopitia, ni mara ngapi unaongea na mtu mwingine kuyahusu? |
| PS2 | In the past month have you talked to anybody about these kinds of problems or worries?  Probe: have you had any sort of meeting or contact with anyone about these sorts of problems or worries? | In the past month have you talked to anybody about these kinds of problems or worries?  Probe: have you had any type of meeting or contact with anyone about these types of problems or worries? | Katika mwezi uliopita umeongea na mtu yeyote kuhusu shida au wasiwasi kama hizi?  Hoji zaidi: umeonekana/kuhudumiwa na mhudumu wa afya au kuwasiliana na mtu yeyote kuhusu shida au wasiwasi kama hizi? | In the past month have you talked to anybody about these kinds of problems or worries?  Probe: have you had any type of meeting or contact with anyone about these types of problems or worries? | Katika mwezi uliopita umeongea na mtu yeyote kuhusu shida au wasiwasi kama hizi?  Hoji zaidi: umeonekana/kuhudumiwa na mhudumu wa afya au kuwasiliana na mtu yeyote kuhusu shida au wasiwasi kama hizi? | In the past month have you talked to anybody about these kinds of problems or worries?  Probe: have you had any type of meeting or contact with anyone about these types of problems or worries? | Katika mwezi uliopita umeongea na mtu yeyote kuhusu shida au wasiwasi kama hizi?  Hoji zaidi: umeonekana/kuhudumiwa na mhudumu wa afya au kuwasiliana na mtu yeyote kuhusu shida au wasiwasi kama hizi? |
| PS3 | Who have you talked to?  Probe: anyone else?" | Who have you talked to?  Probe: anyone else?" | Je! Kuna mtu yeyote umezungumza naye?  Hoji zaidi: kuna mwingine pia? | Who have you talked to?  Probe: anyone else? | Je! Kuna mtu yeyote umezungumza naye?  Hoji zaidi: kuna mwingine pia? | Who have you talked to?  Probe: anyone else? | Je! Kuna mtu yeyote umeongea naye?  Hoji zaidi: kuna mwingine pia? |
| PS4 | In the past month, how often did your parents/guardians understand your problems and worries? | In the past month, how often did your parents/guardians understand your problems and worries? | Katika mwezi uliopita, ni mara ngapi wazazi/walezi wako walielewa shida na wasiwasi wako? | In the past month, how often did your parents/guardians understand your problems and worries? | Katika mwezi uliopita, ni mara ngapi wazazi/walezi wako walielewa shida na wasiwasi wako? | In the past month, how often did your parents/guardians understand your problems and worries? | Katika mwezi uliopita, ni mara ngapi wazazi/walezi wako walielewa shida na wasiwasi wako? |
| SU2 | In your whole lifetime, how often have you thought about ending your life? | We have just a few more questions. Now I want you to think about your whole life. How often have you thought about ending your life? | Tuna maswali machache yaliyobaki. Sasa ningependa ufikirie kuhusu maisha yako yote. Ni mara ngapi umefikiria kujitoa uhai? | We have just a few more questions. Now I want you to think about your whole life. How often have you thought about ending your life? | Tuna maswali machache yaliyobaki. Sasa ningependa ufikirie kuhusu maisha yako yote. Ni mara ngapi umefikiria kujitoa uhai? | We have just a few more questions. Now I want you to think about your whole life. How often have you thought about killing yourself? | Tuna maswali machache yaliyobaki. Sasa ningependa ufikirie kuhusu maisha yako yote. Ni mara ngapi umefikiria kujitoa uhai? |
| SU3 | In your whole lifetime, how often have you ever wished you were dead or wished that your life would be over? For instance, that you could go to sleep and not wake up? | In your whole life, how often have you wished that your life would be over or that you would go to sleep and never wake up? | Katika maisha yako yote, ni mara ngapi umetamani maisha yako yaishe au uende kulala na usiamke tena? | In your whole life, how often have you wished that your life would be over or that you would go to sleep and never wake up? | Katika maisha yako yote, ni mara ngapi umetamani maisha yako yaishe au uende kulala na usiamke tena? | In your whole life, how often have you wished that your life would be over or that you would go to sleep and never wake up? | Katika maisha yako yote, ni mara ngapi umetamani maisha yako yaishe au uende kulala na usiamke tena? |
| SU5 | In the last 12 months, have you tried to end your life, for example, taking poison, hanging yourself, or jumping? | In the last 12 months, have you tried to end your life? For example, by taking poison, hanging yourself, or something else? | Katika miezi kumi na miwili (12) iliyopita, umewahi jaribu kujitoa uhai? Kwa mfano, kwa kunywa sumu, kujinyonga, au kitu kingine? | In the last 12 months, have you tried to end your life? For example, by taking poison, hanging yourself, or something else? | Katika miezi kumi na miwili (12) iliyopita, umewahi jaribu kujitoa uhai? Kwa mfano, kwa kunywa sumu, kujinyonga, au kitu kingine? | In the last 12 months, have you tried to end your life? For example, by taking poison, hanging yourself, or something else? | Katika miezi kumi na miwili (12) iliyopita, umewahi jaribu kujitoa uhai? Kwa mfano, kwa kunywa sumu, kujinyonga, au kitu kingine? |
| SU6 | I want to ask you about the last time you tried. Please tell me which of the following best describes your thinking at the time?  1) it was a serious attempt and I really wanted to die  2) I didn’t care if the plan was going to work or not  3) I didn’t want to die at all; I wanted people to help me | I want to ask you about the last time you tried. Please tell me which of the following best describes your thinking at the time?  1) it was a serious attempt and I really wanted to die  2) I didn’t care if the plan was going to work or not  3) I didn’t want to die at all; I wanted people to help me" | Ningependa kukuuliza kuhusu mara ya mwisho ulijaribu. Tafadhali niambie ni lipi kati ya zifuatazo linaeleza vyema fikira zako wakati huo?  1) lilikuwa jaribio zito na nilitamani sana kufa  2) sikujali kama mpango ungefaulu au la  3) sikutaka kufa kamwe, nilitaka watu wanisaidie | I want to ask you about the last time you tried. Please tell me which of the following best describes your thinking at the time?  1) it was a serious attempt and I really wanted to die  2) I didn’t care if the plan was going to work or not  3) I didn’t want to die at all; I wanted people to help me | Ningependa kukuuliza kuhusu mara ya mwisho ulijaribu. Tafadhali niambie ni lipi kati ya zifuatazo linaeleza vyema fikira zako wakati huo?  1) lilikuwa jaribio zito na nilitamani sana kufa  2) sikujali kama mpango ungefaulu au la  3) sikutaka kufa kamwe, nilitaka watu wanisaidie | I want to ask you about the last time you tried. Please tell me which of the following best describes your thinking at the time?  1) it was a serious attempt and I really wanted to die  2) I didn’t care if the plan was going to work or not  3) I didn’t want to die at all; I wanted people to help me" | Ningependa kukuuliza kuhusu mara ya mwisho ulijaribu. Tafadhali niambie ni lipi kati ya zifuatazo linaeleza vyema fikira zako wakati huo?  1) lilikuwa jaribio zito na nilitamani sana kufa  2) sikujali kama mpango ungefaulu au la  3) sikutaka kufa kamwe, nilitaka watu wanisaidie |
| SU7 | In the past 2 weeks, have you thought about ending your life? | In the past 2 weeks, have you thought about ending your life? | Katika wiki mbili (2) zilizopita, umefikiria kuhusu kujitoa uhai? | In the past 2 weeks, have you thought about ending your life? | Katika wiki mbili (2) zilizopita, umefikiria kuhusu kujitoa uhai? | In the past 2 weeks, have you thought about ending your life? | Katika wiki mbili (2) zilizopita, umefikiria kuhusu kujitoa uhai? |
| Su8 | Do you want to end your life right now? | Do you want to end your life right now? | Je! Unataka kujitoa uhai sasa hivi? | Do you want to end your life right now? | Je! Unataka kujitoa uhai sasa hivi? | Do you want to end your life right now? | Je! Unataka kujitoa uhai sasa hivi? |
| **RCADS response options** | | | | | | | |
| 3 | Always | Always | Kila wakati | All the time | Kila wakati | All the time | Kila wakati |
| 2 | Often | Often | Mara nyingi | Many times | Mara nyingi | Many times | Mara nyingi |
| 1 | Sometimes | Sometimes | Mara kwa mara | Sometimes | Mara kwa mara | Sometimes | Mara kwa mara |
| 0 | Never | Never | Sipati kabisa | Never | Sipati kabisa | Never | Sipati kabisa |
|  |  |  |  |  |  |  |  |
| **Patient Health Questionnaire- 9 (PHQ-9)** | | | | | | | |
| 1 | Little interest or pleasure in doing things | Little interest or less happiness in daily activities | Kupoteza hamu au furaha katika shughuli za kila siku | Little interest or pleasure in doing things | Kupoteza hamu au furaha katika shughuli za kila siku | Little interest or less happiness in daily activities | Kupoteza hamu au furaha katika shughuli za kila siku |
| 2 | Feeling down, depressed, or hopeless | Feeling bored, depressed, or hopeless | Kukosa furaha/kuboeka, mawazo mengi, au kukosa tumaini | Feeling down, depressed, or hopeless | Kukosa furaha/kuboeka, mawazo mengi, au kukosa tumaini | Feeling bored, depressed, or hopeless | Kukosa furaha/kuboeka, mawazo mengi, au kukosa tumaini |
| 3 | Trouble falling or staying asleep, or sleeping too much | Trouble falling or staying asleep, or sleeping too much | Shida ya kupata usingizi au kulala sana | Trouble falling or staying asleep, or sleeping too much | Shida ya kupata usingizi au kulala sana | Trouble falling or staying asleep, or sleeping too much | Shida ya kupata usingizi au kulala sana |
| 4 | Feeling tired or having little energy | Feeling tired or having little energy | Kusikia uchovu au kuwa na nguvu kidogo | Feeling tired or having little energy | Kusikia uchovu au kuwa na nguvu kidogo | Feeling tired or having little energy | Kusikia uchovu au kuwa na nguvu kidogo |
| 5 | Poor appetite or overeating | Poor appetite or overeating | Kukosa hamu ya kula au kula sana | Poor appetite or overeating | Kukosa hamu ya kula au kula sana | Poor appetite or overeating | Kukosa hamu ya kula au kula sana |
| 6 | Feeling bad about yourself- or that you are a failure or have let yourself or your family down | Feeling bad or as a failure about yourself or a disappointment to your family | Kusikia vibaya au kwamba umeshindwa au umeaibisha familia yako | Feeling bad about yourself- or that you are a failure or have let yourself or your family down | Kusikia vibaya au kwamba umeshindwa au umeaibisha familia yako | Feeling bad or as a failure about yourself or a disappointment to your family | Kusikia vibaya au kwamba umeshindwa au umeaibisha familia yako |
| 7 | Trouble concentrating on things, such as reading the newspaper or watching television | Trouble/problem focusing on things, such as starting and finishing activities e.g. home duties | Shida ya kuzingatia/kuwa makini kwa kazi yoyote nyumbani | Trouble concentrating on things, such as reading the newspaper or watching television | Shida ya kuzingatia/kuwa makini kwa kazi yoyote nyumbani | Trouble/problem focusing on things, such as starting and finishing activities e.g. home duties | Shida ya kuzingatia/kuwa makini kwa kazi yoyote nyumbani |
| 8 | Moving or speaking so slowly that other people could have noticed. Or the opposite- being fidgety or restless that you have been moving around a lot more than usual | Moving or talking so slowly that other people could have noticed. Or being unable to relax as usual | Kutembea au kuongea polepole sana kwamba watu wengine wangeweza kugundua. Au kutokuwa na uwezo wa kutulia kama kawaida | Moving or speaking so slowly that other people could have noticed. Or the opposite- being fidgety or restless that you have been moving around a lot more than usual | Kutembea au kuongea polepole sana kwamba watu wengine wangeweza kugundua. Au kutokuwa na uwezo wa kutulia kama kawaida | Moving or talking so slowly that other people could have noticed. Or being unable to relax as usual | Kutembea au kuongea polepole sana kwamba watu wengine wangeweza kugundua. Au kutokuwa na uwezo wa kutulia kama kawaida |
| 9 | Thoughts that you would be better off dead, or of hurting yourself | Thoughts that you would prefer being dead, or of hurting yourself | Mawazo kwamba ungependelea kufa, au kujiumiza | Thoughts that you would be better off dead, or of hurting yourself | Mawazo kwamba ungependelea kufa, au kujiumiza | Thoughts that you would prefer being dead, or of hurting yourself | Mawazo kwamba ungependelea kufa, au kujiumiza |
| **PHQ-9 response options** | | | | | | | |
| 3 | Nearly every day | Nearly every day | Karibu kila siku | Nearly every day | Karibu kila siku | Nearly every day | Karibu kila siku |
| 2 | More than half the days | More than seven days | Zaidi ya siku saba | More than seven days | Zaidi ya siku saba | More than seven days | Zaidi ya siku saba |
| 1 | Several days | Several days | Siku kadhaa | Several days | Siku kadhaa | Several days | Siku kadhaa |
| 0 | Not at all | Not at all | Hapana kabisa | Not at all | Hapana kabisa | Not at all | Hapana kabisa |
|  |  |  |  |  |  |  |  |
| 10 | If you checked off any problems, how difficult have these problems made it for you to do your work, take care of things at home, or get along with other people? | If you have experienced any problems, how difficult have these problems made it for you to do your work, take care of things at home, or get along with other people? | Ikiwa umepatwa na shida yoyote, ni vipi shida hizi zimekusababishia ugumu kufanya kazi yako, kutunza vitu nyumbani, au kushirikiana vizuri na watu wengine? | If you checked off any problems, how difficult have these problems made it for you to do your work, take care of things at home, or get along with other people? | Ikiwa umepatwa na shida yoyote, ni vipi shida hizi zimekusababishia ugumu kufanya kazi yako, kutunza vitu nyumbani, au kushirikiana vizuri na watu wengine? | If you have experienced any problems, how difficult have these problems made it for you to do your work, take care of things at home, or get along with other people? | Ikiwa umepatwa na shida yoyote, ni vipi shida hizi zimekusababishia ugumu kufanya kazi yako, kutunza vitu nyumbani, au kushirikiana vizuri na watu wengine? |
| **PHQ-9 difficulty item response options** | | | | | | | |
|  | Extremely difficult | Extremely difficult | Vigumu kupita kiasi | Extremely difficult | Vigumu kupita kiasi | Extremely difficult | Vigumu kupita kiasi |
|  | Very difficult | Very difficult | Vigumu sana | Very difficult | Vigumu sana | Very difficult | Vigumu sana |
|  | Somewhat difficult | Slightly difficult | Vigumu kiasi | Slightly difficult | Vigumu kiasi | Slightly difficult | Vigumu kiasi |
|  | Not difficult at all | Not difficult at all | Sio vigumu kabisa | Not difficult at all | Sio vigumu kabisa | Not difficult at all | Sio vigumu kabisa |
