# Supplementary material for: Cultural and contextual adaptation of mental health measures in Kenya: An adolescent-centered transcultural adaptation of measures study
Source: PLoS One. 2022 Dec 15;17(12):e0277619. doi: 10.1371/journal.pone.0277619 (PMC9754261; doi:10.1371/journal.pone.0277619)
Supplement: Attachment — Submitted filename: Response to reviewers.docx [file pone.0277619.s003.docx]

  30^th^ August 2022

To

The Editor

PLOS ONE

**Re**: *Resubmission of paper ‘*PONE-D-22-12888 *Cultural and contextual adaptation of mental health measures in Kenya: an adolescent-centered transcultural adaptation of measures study’*

Dear Dr. Caroline Kingori

We want to thank you for reviewing our manuscript and we have spent some time editing the paper and have provided point- by-point response given here below. We are grateful to the reviewers for their comments and feedback.

We hope you the edited paper will meet your expectation.

Regards

Vincent Nyongesa

Manasi Kumar

**Response to reviewers**

Journal Requirements:

When submitting your revision, we need you to address these additional requirements.

1. Please ensure that your manuscript meets PLOS ONE's style requirements, including those for file naming. The PLOS ONE style templates can be found at

<https://journals.plos.org/plosone/s/file?id=wjVg/PLOSOne_formatting_sample_main_body.pdf> and

<https://journals.plos.org/plosone/s/file?id=ba62/PLOSOne_formatting_sample_title_authors_affiliations.pdf>

***Response:*** *we thank you and have now edited the manuscripts per guidance.*

2. You indicated that you had ethical approval for your study. In your Methods section, please ensure you have also stated whether you obtained consent from parents or guardians of the minors included in the study or whether the research ethics committee or IRB specifically waived the need for their consent.

***Response****: Thank you, we have added this statement (Please see page 9)*

3. We note that the grant information you provided in the ‘Funding Information’ and ‘Financial Disclosure’ sections do not match.

When you resubmit, please ensure that you provide the correct grant numbers for the awards you received for your study in the ‘Funding Information’ section.

***Response:*** *we thank for noticing this inconsistency and we have now addressed this. The senior author was funded by Fogarty and the costs of activities were partially covered by UNICEF.*

4. We note that you have indicated that data from this study are available upon request. PLOS only allows data to be available upon request if there are legal or ethical restrictions on sharing data publicly. For more information on unacceptable data access restrictions, please see <http://journals.plos.org/plosone/s/data-availability#loc-unacceptable-data-access-restrictions>.

In your revised cover letter, please address the following prompts:

a) If there are ethical or legal restrictions on sharing a de-identified data set, please explain them in detail (e.g., data contain potentially sensitive information, data are owned by a third-party organization, etc.) and who has imposed them (e.g., an ethics committee). Please also provide contact information for a data access committee, ethics committee, or other institutional body to which data requests may be sent.

b) If there are no restrictions, please upload the minimal anonymized data set necessary to replicate your study findings as either Supporting Information files or to a stable, public repository and provide us with the relevant URLs, DOIs, or accession numbers. For a list of acceptable repositories, please see <http://journals.plos.org/plosone/s/data-availability#loc-recommended-repositories>.

We will update your Data Availability statement on your behalf to reflect the information you provide.

***Response:*** *We have attached focus group discussion, and cognitive interview transcripts, and a table as supplementary information*

5. Your ethics statement should only appear in the Methods section of your manuscript. If your ethics statement is written in any section besides the Methods, please delete it from any other section.

***Response:*** *Thank you, we have corrected this now.*

5. Review Comments to the Author

Please use the space provided to explain your answers to the questions above. You may also include additional comments for the author, including concerns about dual publication, research ethics, or publication ethics. (Please upload your review as an attachment if it exceeds 20,000 characters)

Reviewer #1: • This was a very good and enjoyable manuscript. The need for culturally appropriate measurement scales is a logical point and well explained in the introduction section. My feedback is really on structure and writing to make sure it is readable and findings easy to review.
• Introduction section – provides a good overview of what the TAA process is and the importance of using a participatory method to improve measurement scales. In the objective, you mention only FGDs (no mention of C.Is).

***Response:*** *Thank you, we have now mentioned Cognitive interviews under this section*

• Box 1 – restructure/reformat table another way as the words seems to scatter off making it difficult to identify the 3 different columns (Domains, FGD & CD findings). Same applies for Table 3

***Response:*** *Thank you, we have added table grids to make it readable*

• Page 31 – 3rd sentence “there are dual roles…” --- reconsider that sentence, reads a bit unclear

***Response*:** *Thank you, we have cut down and restructured this sentence*

• Study implications are very well discussed though the latter discussion read a bit disconnected from the overall scope of the study. This last paragraph focuses, though accurate, the need for support services. The study findings tended to focus on the actual adaptation of the scales and not on the lived experience/challenges of the youth. Though important, in order for this to connect with this last paragraph, maybe providing some findings on that so that we can get context on what the service needs are.

***Response*:** *Thank you for pointing at this, we have clarified this section (See page 32)*

• Writing – a few scattered grammatical errors noted (either missing a word or have an extra work somewhere. Be sure to review entire document to catch those hidden errors.

***Response*:** *Thank you for pointing to this, we have checked this on Grammarly software and edited the work*

Reviewer #2: This study described the process of culturally adapting mental health assessment measures for adolescents in Kenya. This article is important because it highlights the lack of culturally tailored mental health tools for this population. The strength of this article is that authors used a thorough multi-stage adaptation process. To improve this article, I suggest that authors should focus on reducing some redundancy in the text and clarifying the methods that were used in the adaptation process.
Introduction
Page 6, Paragraph 1: This paragraph is quite redundant. The following four sentences say very similar things and can be combined.
• In addition, multi-stakeholder inquiries involving representatives from different groups in the society are crucial as they bring different views that help tailor the tools to meet specific needs of the target group (13).
• Global mental health emphasizes multistakeholder engagement and recognizes the need for linguistic and cultural adaptation of mental health tools (15,16).
• Therefore, involvement of service consumers or community members in making decisions during cultural adaptations and letting experts make sure final changes and decisions are inclusive helps maintain touch with the local realities (17).
• Checking with subject experts and end-users from time to time ensures relevance of the adapted tool.
Methods

***Response:*** *Thank you, we have combined and edited the sentences per your suggestion (See page 6)*

• The paragraphs for study setting and study site should be combined

***Response:*** *Thank you for pointing to this, we now have merged them (Please see page 7)*

• It was hard to follow all of the data collection activities that were conducted and how they were ordered. To clarify this section, clearly define what the TTA approach is. Additionally, provide greater details about the protocols for each data collection activity.

***Response*:** *Thank you, we have elaborated the steps, and this work has been explained in the following publications we have now added more information here (Please see page 8).*

Joseph Hayes, Liliana Carvajal, Zeinab Hijazi, Jill Witney Ahs, P. Murali Doraiswamy, Fatima Azzahra El Azzouzi, Cameron Fox, Helen Herrman, Charlotte Petri Gornitzka, Brandon Staglin, Miranda Wolpert,You Can’t Manage What You Do Not Measure - Why Adolescent Mental Health Monitoring Matters, Journal of Adolescent Health, 2021, ISSN 1054-139X, https://doi.org/10.1016/j.jadohealth.2021.04.024.(https://www.sciencedirect.com/science/article/pii/S1054139X21002214)

Carvajal L, Ottman K, Ahs JW, Li GN, Simmons J, Chorpita B, Requejo JH, Kohrt BA. Translation and Adaptation of the Revised Children's Anxiety and Depression Scale: A Qualitative Study in Belize. J Adolesc Health. 2022 Aug 4:S1054-139X(22)00494-3. doi: 10.1016/j.jadohealth.2022.05.026. Epub ahead of print. PMID: 35934586.

Liliana Carvajal, Jill W. Ahs, Jennifer Harris Requejo, Christian Kieling, Andreas Lundin, Manasi Kumar, Nagendra P. Luitel, Marguerite Marlow, Sarah Skeen, Mark Tomlinson, Brandon A. Kohrt, Measurement of Mental Health Among Adolescents at the Population Level: A Multicountry Protocol for Adaptation and Validation of Mental Health Measures, Journal of Adolescent Health,2022,ISSN 1054-139X,https://doi.org/10.1016/j.jadohealth.2021.11.035. (<https://www.sciencedirect.com/science/article/pii/S1054139X21006935>)

• In analysis section, describe how data from body mapping and free listing activity were analyzed if differently from CI and FGD transcripts.

**Response:** *Thank you, the data from body mapping and free listing were part of FGDs, thus analyzed together*

Results
• It is mentioned that pregnant and parenting adolescents were included as participants, however the extent to which their responses differed from non-parenting adolescents isn’t included in the results section.

***Response*:** *Thank you for pointing to this, however, their responses were not so different from the non-parenting ones, they expressed more problems that come with conceiving at a younger age and challenges associated with continuation of education.*

• It was helpful to see the table with all of the adjustments that were made, but the table was hard to follow. This is partially due to formatting. It may be more useful to have that table as an appendix and include the final items in Kiswahili and English in the main text.

***Response:*** *Thank you, we have added table grids for easy readability, we have also provided supplementary material with final wording.*

• Page 28-29: The section, “Age and gender differences around idioms used to express mental health difficulties and distress” is interesting, but it is unclear which parts of data collection the findings come from. It seems like a mixture of observations and the body mapping exercise. Additionally, parts of this section also are more appropriate for the discussion than the results section. For example, the sentences explaining what Sheng is should be in the discussion section.

***Response*:** *Thank you, we have now clarified this on page 29, we have also moved part of this paragraph to discussion per your suggestions*

Discussion
• The discussion notes that the study sought to gain an improved understanding of the “UNICEF mental health module for adolescents,” or “MMA protocol” yet that was not described in the methods and results. Include information in the methods describing what the module was, the methods for assessing the module, and participants assessment of the module.

***Response*:** *the UNICEF publications came after the submission were made. We have now annotated those and reflected on some of these issues in the methods section*.

• Include a paragraph where you describe any study limitations and strengths.

**Response:** *Thank you for this suggestion, we have included this section (See page 32)*
